# Effects of Arbutin on Potassium Bromate-Induced Erythrocyte Toxicity in Rats: Biochemical Evaluation of Some Metabolic Enzyme Activities In Vivo and In Vitro

**DOI:** 10.1021/acsomega.3c06101

**Published:** 2023-09-22

**Authors:** Yusuf Temel

**Affiliations:** † Solhan Health Services Vocational School, Bingöl University, Bingöl12000, Turkey; ‡ Faculty of Arts and Sciences, Bingol University, Bingol12000, Turkiye

## Abstract

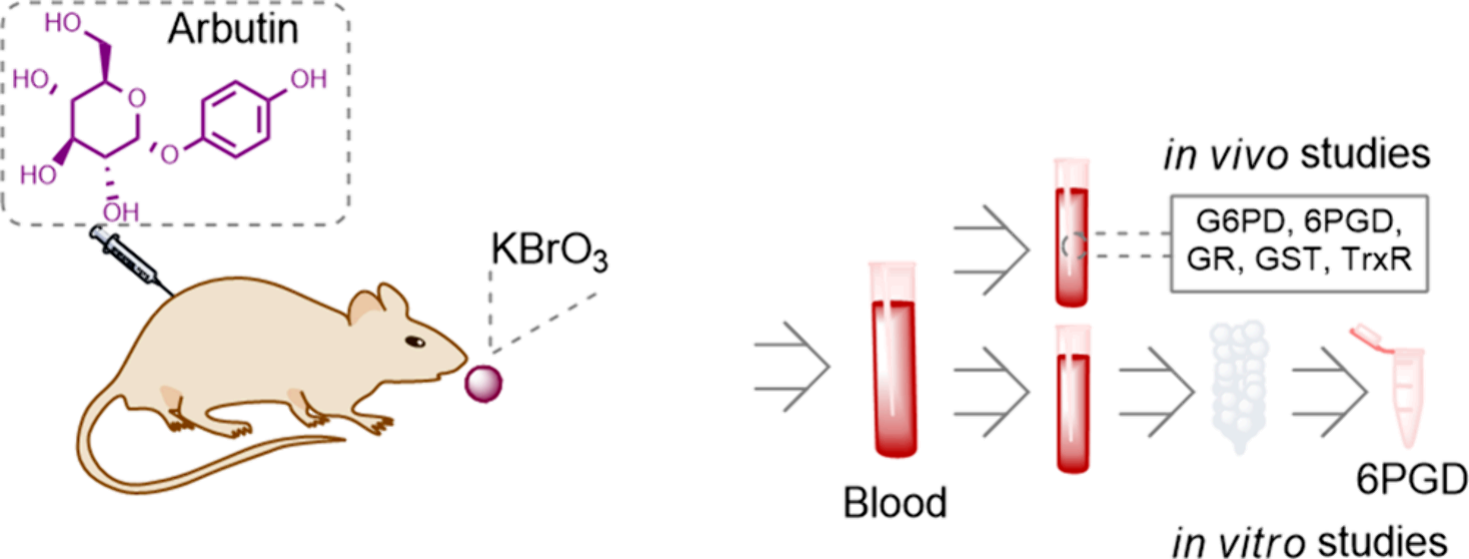

In the present study, the inhibitory effect of potassium bromate on the pentose phosphate pathway and intracellular antioxidant systems enzymes (glucose 6-phosphate dehydrogenase (G6PD), 6-phosphogluconate dehydrogenase (6PGD), glutathione reductase (GR), glutathione S-transferase (GST), and thioredoxin reductase (TrxR)) and the role of arbutin in ameliorating this inhibition were investigated. In the in vivo phase of the study, Wistar Albino rats (28 male adults) were randomly divided into four groups. Control (*n* = 7): isotonic serum (0.5 mL, i.p), potassium bromate group (*n* = 7): potassium bromate (100 mg/kg), arbutin group (*n* = 7): arbutin (i.p.) (50 mg/kg/day), potassium bromate + arbutin, and Group (*n* = 7): potassium bromate (100 mg/kg) + arbutin (50 mg/kg/day) (i.p). The results of in vivo study showed that the activities of G6PD, 6PGD, GR, and TrxR enzymes were strongly inhibited in potassium bromate groups (*p* < 0.05). It was determined that GST enzyme activity decreased in the potassium bromate group, but this decrease was not statistically significant compared to the control group (*p* ⩾ 0.05). A statistically significant increase was found in G6PD, 6PGD, GST, and TrxR enzyme activities in the arbutin group compared to the control group (*p* < 0.05). The increase in GR enzyme activity was not statistically significant (*p* ⩾ 0.05). The potassium bromate + arbutin group’s enzyme activity increased in comparison to the potassium bromate group and was discovered to be closer to the control group. It was found that potassium bromate inhibited the 6PGD enzyme obtained from rat erythrocyte tissues with IC_50_ = 346 μM value and *K*_i_ = 434.4 μM ± 6.1 value, and the inhibition was noncompetitive.

## Introduction

Potassium bromate (KBrO_3_) is commonly used in cosmetics, industry, and as a food additive owing to its oxidizing abilities. In cosmetics, it serves as a neutralizer in cold wave hair treatments, in the oxidation of sulfur and boat dyes in industry, and as a food additive thanks to its flexibility and strength in dough. It is also used in brewing, cheese production, and the pharmaceutical industry.^1,2^ In the process of disinfecting drinking water with ozonation, bromate also presents as a significant byproduct.^3^ Research has demonstrated that potassium bromate reduces the amount of niacin and key vitamins A, B, and E during the bread-making process, which has a negative effect on the nutritional value of bread. The studies have also demonstrated that potassium bromate may result in renal failure, deafness, skin redness, and eye pain, particularly cancer.^1,4,5^

Arbutin (C_12_H_16_O_7_), a hydrophilic polyphenol, has two isomers, including (p-hydroxyphenyl-β-d-glucopyranoside) and alpha-arbutin (4-hydroxyphenyl-α-glucopyranoside) and β-arbutin (4-hydroxyphenyl-β-glucopyranoside).^6^ It is abundant in food plants such as wheat, broccoli, pepper, fruits, and coffee. It can also be obtained from a variety of synthetic sources, including metabolic engineering of microorganisms.^7^ Arbutin, a hydroquinone glycoside, is used in cosmetics and medications for a variety of biological purposes according to recent studies. It is used in cosmetics as a skin-lightening substance and antiaging agent. In the pharmaceutical field, it has been shown that it is effective in the treatment of several disorders.^7^

The pentose phosphate pathway has two key functions in cells: it generates ribose 5-phosphate, which is required for the synthesis of DNA and RNA, as well as NADPH (nicotinamide adenine dinucleotide phosphate in its reduced form). NADPH synthesis is critical for the defense of cells against oxidative stress since it functions as an electron donor for numerous enzymatic processes necessary in biosynthetic pathways. The pentose phosphate pathway’s initial step is catalyzed by the enzyme glucose-6-phosphate dehydrogenase (G6PD), supplying all cells with acts a reducing power in the form of NADPH.^8−10^ 6-phosphogluconate dehydrogenase (6PGD), an essential enzyme for biological systems, catalyzes the conversion of 6-phosphogluconate into D-ribulose 5-phosphate, resulting in the production of NADPH in the presence of NADP^+^.^11−13^ The glutathione reductase (GR) enzyme is required for glutathione disulfide (GSSG) to be converted to its reduced form (GSH). GSH is necessary for the protection of cells against oxidative stress as an antioxidant. Moreover, GSH functions as a cofactor in isomerization activities, a reaction partner for the detoxification of xenobiotics, and a storage and transport mechanism for cysteine.^14−16^ Glutathione-S transferases (GSTs) are known as Phase II detoxification enzymes. GSTs are found in most living things and are essential for maintaining cellular homeostasis.^17,18^ GSTs primarily have a cytoprotective role by facilitating the reaction that turns reactive electrophiles produced by cytochrome P450 metabolism into GSH conjugates.^19^ Thioredoxine reductases (TrxRs) play a crucial role in the generation of deoxyribonucleotides for DNA synthesis, redox regulation of cell activity, and antioxidant defense.^20,21^

There was no study found in the literature on the protective effect of arbutin on potassium bromate-induced rat enzyme activity in erythrocyte cells. The aim of this study was to investigate the inhibitory effects of potassium bromate on G6PD, 6PGD, GR, GST, and TrxR enzymes in rat erythrocyte cells and determine the potential of arbutin, a natural antioxidant, to reduce these detrimental effects in vivo and in vitro.

## Materials and Methods

### Chemicals

Standard serum albumin, 5,5′-Dithiobis(2-nitrobenzoic acid) (DTNB), potassium bromate, arbutin, G6P, 6PGA, Tris, NADP+, protein assay reagent, NADPH, electrophoresis chemicals, and 2′,5′-ADP Sepharose-4B were obtained from Merck or Sigma-Aldrich (St. Louis, MO, USA).

### Experimental Design

Four groups composed of 28 mature Wistar albino male rats were assigned at random. The toxic dose of potassium bromate (KBrO_3_) was established according to a previous study.^3^ The experiment carried out by Zolfalipor et al. served as the basis for the therapeutic dose of arbutin.^22^ During 5 days, intraperitoneal injections of sterile saline (0.9% NaCl) were given to the control group. The KBrO_3_ group received a single dose (100 mg/kg) of KBrO_3_ (gavage). The arbutin group received 50 mg/kg bw/day (i.p.) for 5 days. The KBrO_3_ and arbutin group was KBrO_3_ 100 mg/kg (gavage) single dose + arbutin (50 mg/kg bw/day) (i.p.) for 5 days. To collect blood samples, the rats were given 50 mg/kg of ketamine hydrochloride and 10 mg/kg of xylazine hydrochloride at the end of the fifth day.

### Determination of the G6PD and 6PGD Enzyme Activities

In this investigation, the Beutler technique was used to evaluate the spectrophotometric activity of the G6PD and 6PGD enzymes at 340 nm wavelengths. The spectrophotometric measurement of the G6PD and 6PGD enzyme activity involved observing the increase in absorbance caused by the decrease of NADP^+^. For the purpose of measuring the activity of G6PD and 6PGD, a cuvette containing 0.5 mM ethylenediaminetetraacetic acid (EDTA), 0.01 mM MgCl_2_, 0.6 mM G6*P*/6PGA, and 0.2 mM NADP^+^ was produced. One mole of NADPH oxidation per minute is the definition of an enzyme unit.^23,24^

### Determination of the GR Enzyme Activity

The Carlberg and Mannervik technique was used to measure GR enzyme activity (1981). The cuvette content consisted of 0.1 mM K-phosphate, 20 mM GSSG, and 2 mM NADPH for the measurement of GR activity. One enzyme unit is defined as 1 μmol of NADPH oxidation per minute.^13,25^

### Determination of the GST Enzyme Activity

The Habig technique was used to gauge the activity of the GST enzyme; 20 mM GSH, 25 mM 1-chloro-2,4-dinitrobenzene (CDNB), 0.1 mM EDTA, and 0.1 M K-phosphate were included in the mixture created for the GST test. The enzyme unit calculated from the conversion of CDNB to DNB-SG at 340 nm.^26,27^

### Determination of the TrxR Enzyme Activity

TrxR enzyme activity was measured using the Holmgren method. TrxR activity was measured using a mixture containing 100 mM K-phosphate, 0.2 mM NADPH, 10 mM EDTA, 0.2 mg/mL bovine serum albumin (BSA), and 5 mM DTNB. The enzyme unit calculated the reduction of DTNB by NADPH at 412 nm per minute.^21,28^

### Preparation of the Hemolysate

Hemolysate was prepared in the current investigation using the Temel et al. procedure. At the end of the fifth day, blood tissue samples were collected from rats killed under anesthesia in accordance with ethical rules. Rat blood samples were transferred to anticoagulant-containing tubes (EDTA). Twenty minutes were centrifuging blood samples at 3500 rpm. Red blood cells were obtained after the plasma was eliminated, and they were then subjected to three washings in a 0.16 molar potassium chloride solution. After every wash, the centrifugation process was repeated. The recovered erythrocytes were then homogenized three times with deionized water. After hemolysis of erythrocytes, centrifugation was performed at +4 °C (30 min, 10,000*g*). Centrifuged samples were stored at −20 °C until the time of analysis.^29−31^

### 2′,5′-ADP Sepharose-4B Affinity Chromatography

One of the most often applied methods for enzyme purification is affinity chromatography. In this study, affinity chromatography was used to purify the 6PGD enzyme. This was accomplished by weighing 2 g of dried 2′, 5′-ADP Sepharose-4B gel for a 10 mL bed volume and washing it with 200 mL of distilled water. The gel was then put onto the column and stabilized in a buffer solution (50 mM KH_2_PO_4_, 1 mM EDTA, 1 mM DTT, pH 7.3). The hemolysate was placed onto the column after the stabilization process was complete. After the absorbance difference at 280 nm reached 0.05, the column was washed with a stabilization buffer. Using 80 mM K-phosphate, 10 mM EDTA, 80 mM KCI, and 0.5 mM NADP^+^, elution was completed (pH 7.3). Every process was performed at +4 °C.

### Animals

Adult Wistar albino male rats (28) were obtained from the Bingol University Experimental Research and Application Center. The rats were between 200 and 300 g and 10–12 weeks of age. The rats were kept in an environment with a 12 h light/dark cycle, a humidity of 45 ± 5%, and a temperature of 25 ± 2 °C. Standard laboratory feed and water were used in the feeds ad libitum. The investigation was initiated after approval of the experimental protocols by the Bingöl University Animal Experiments Local Ethics Committee (BUHADEK: 12.07.2023-E.113967).

### In Vitro Effect of Potassium Bromate and Arbutin

Different concentrations (118–708 M) of potassium bromate ions (KBrO_3_) were included in the reaction medium, which was composed of 6PGA and NADP^+^ substrates, in order to assess the impact of potassium bromate ions on the 6PGD enzyme purified from rat erythrocytes by 2′-5′ ADP sepharose affinity chromatography. Three times each of the measurements was taken to statistically analyze the data. Measurements other than that of the inhibitor (100% activity) were preferred for the control process. The IC_50_ values (the inhibitor concentration that decreases enzyme activity by half) were calculated in accordance with an activity%-inhibitor concentration diagram created with Microsoft Office Excel 2010. Lineweaver–Burk plots were created using 5 different substrate concentrations (6PGA) and 3 different inhibitor concentrations (KBrO_3_). The value of *K*_i_ (the inhibition constant) was determined using Lineweaver–Burk graphics.

### Analysis of Kinetic Data

The SPSS Statistics 20 program was used for the statistical analysis. The data were presented as mean ± SD. To compare the differences between the groups, one-way ANOVA and Tukey’s post hoc least significant difference (LSD) were utilized. When the *p* value was <0.05, the difference between the groups was deemed significant. In the in vitro analysis, Microsoft Excel 2010 was used.

## Results

**Figure 1:**
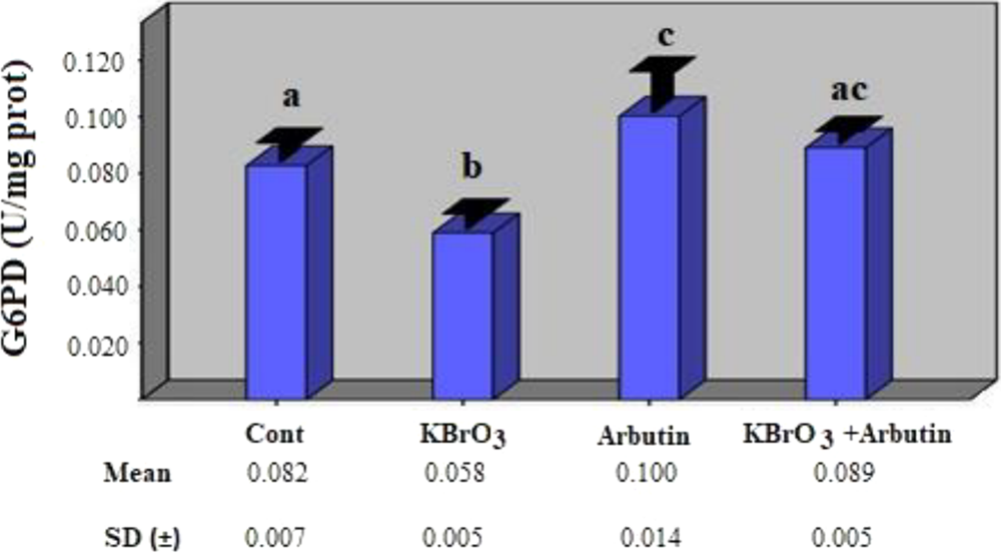
Effects of potassium bromate and arbutin on the activity of the G6PD enzyme in rat erythrocytes in vivo*.* Different letters in (a–c) represent statistical differences between the groups (*p* < 0.05).

In this study, the toxic effect of potassium bromate on rat erythrocyte metabolic enzymes and the role of arbutin in reducing this effect were investigated. According to study findings, the potassium bromate group’s G6PD enzyme activity was statistically substantially lower than that of the control group (*p* < 0.05). When compared to the control group, it was shown that the G6PD enzyme activity increased statistically significantly in the arbutin group (*p* < 0.05). In the potassium bromate + arbutin group, however, it was determined that the G6PD enzyme activity increased compared to the potassium bromate group and approached the control group, and there was no statistically significant difference when compared with the control group (Figure 1) (*p* > 0.05).

**Figure 2:**
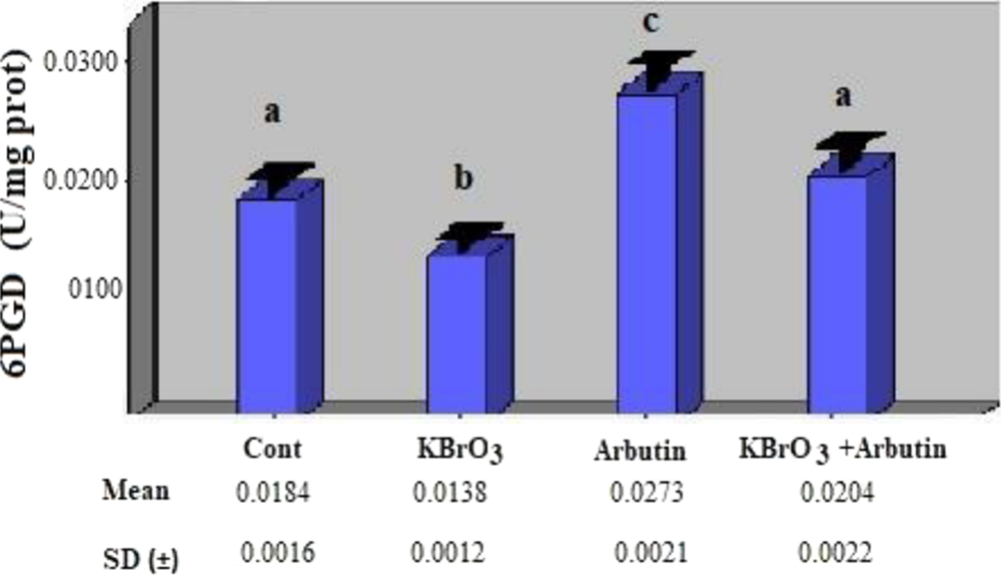
Effects of potassium bromate and arbutin on the activity of the 6PGD enzyme in rat erythrocytes in vivo*.* Different letters in (a–c) represent statistical differences between the groups (*p* < 0.05).

The effects of these treatments were also assessed on 6PGD enzyme activity, and it was shown that the potassium bromate group’s activity was statistically considerably lower than the control group’s (*p* < 0.05). When compared to the control group, there was a statistically significant increase in the 6PGD enzyme activity in the arbutin group (*p* < 0.05). In the potassium bromate + arbutin group, when compared to the potassium bromate group, the 6PGD enzyme activity rose and reached that of the control group, and there was no significant difference between the two groups (Figure 2) (*p* > 0.05).

**Figure 3:**
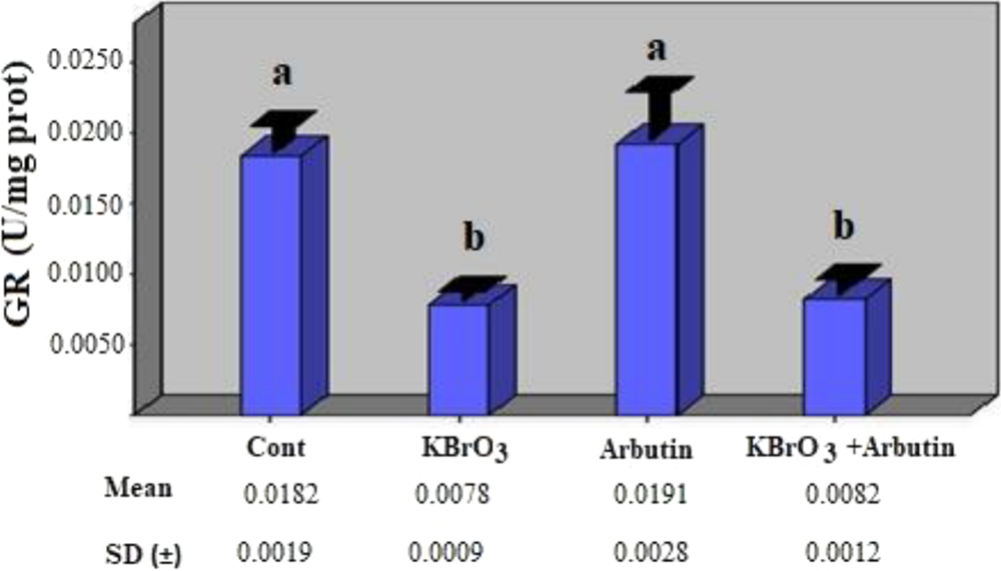
Effects of potassium bromate and arbutin on the activity of the GR enzyme in rat erythrocytes in vivo*.* Different letters in (a, b) represent statistical differences between the groups (*p* < 0.05).

The effects of these treatments were also assessed on GR enzyme activity; it was shown that the potassium bromate group had much lower enzyme activity than the control group, and that this difference in activity was statistically significant (*p* < 0.05). The GR enzyme activity in the arbutin group was found to be comparable to that of the control group, and the difference in activity was not statistically significant (*p* > 0.05). When compared to the potassium bromate group, the potassium bromate + arbutin group showed a statistically negligible increase in GR enzyme activity. In comparison to the control group, a statistically significant drop in GR enzyme activity was seen in the potassium bromate + arbutin group (Figure 3) (*p* < 0.05).

**Figure 4:**
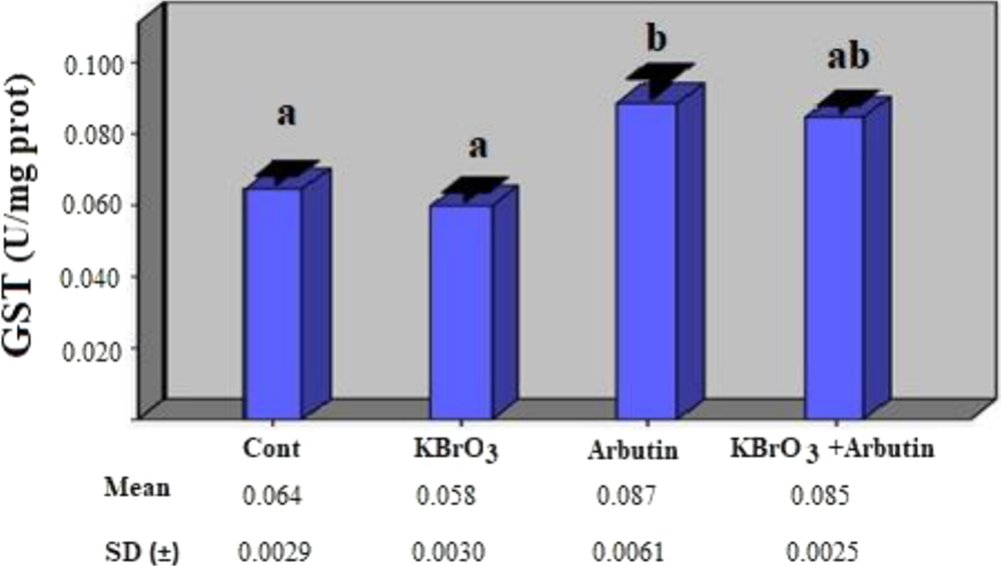
Effects of potassium bromate and arbutin on the activity of the GST enzyme in rat erythrocytes in vivo. Different letters in (a, b) represent statistical differences between the groups (*p* < 0.05).

When the activity of the GST enzyme was measured, a statistically negligible drop in enzyme activity was discovered between the potassium bromate group and the control group (*p* > 0.05). It was determined that there was a statistically significant increase in GST enzyme activity in the arbutin group compared to the control group (*p* < 0.05). In the potassium bromate + arbutin group, the enzyme activity was decreased compared to the arbutin group, and a statistically insignificant increase in GST enzyme activity was detected when compared to the control group (Figure 4) (*p* > 0.05).

**Figure 5:**
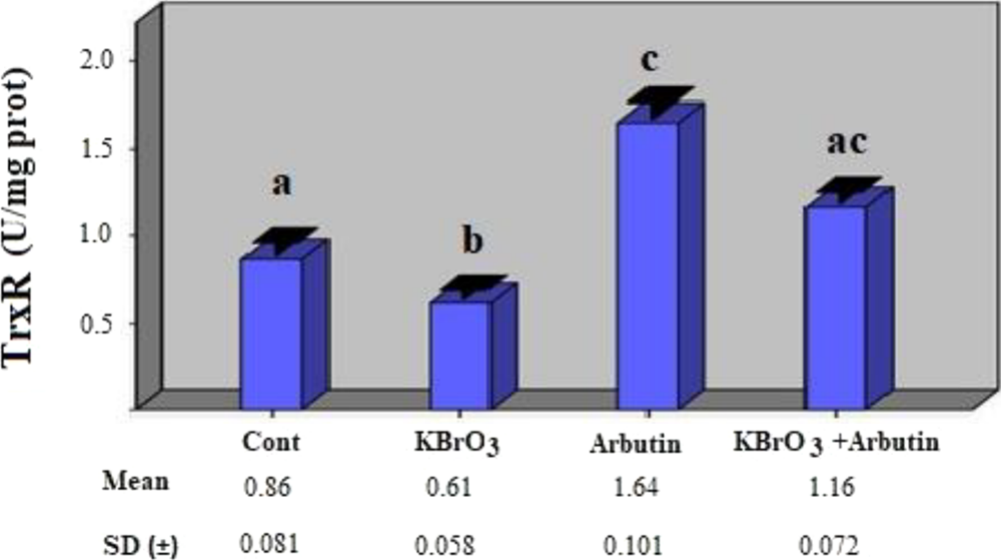
Effects of potassium bromate and arbutin on the activity of the TrxR enzyme in rat erythrocytes in vivo. Different letters in (a–c) represent statistical differences between the groups (*p* < 0.05).

The effects of these treatments were also assessed on TrxR enzyme activity, and it was discovered that there was a statistically significant drop in enzyme activity in the potassium bromate group compared to the control group (*p* > 0.05). TrxR enzyme activity was shown to be higher in the arbutin group compared to the control group (*p* < 0.05). The enzyme activity was found to be lower in the potassium bromate + arbutin group compared to the arbutin group, and the TrxR enzyme activity was shown to be statistically significantly higher when compared to the control group (Figure 5) (*p* < 0.05).

**Figure 6:**
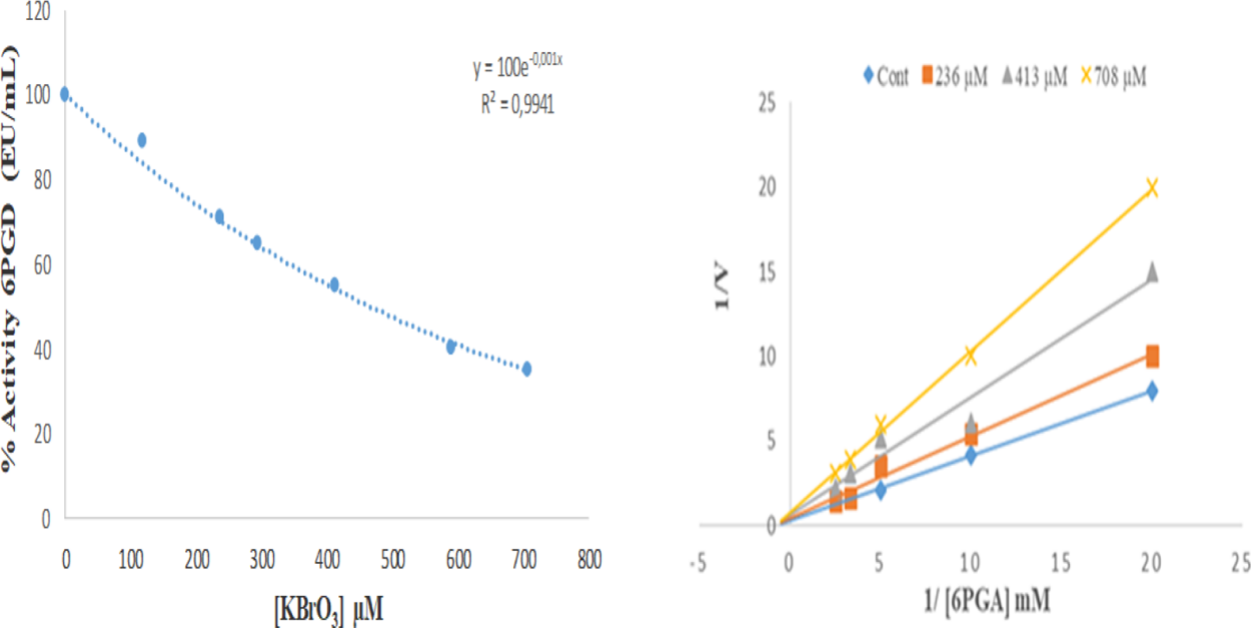
In vitro effect of potassium bromate ions on the rat erythrocyte 6PGD enzyme (IC_50_ and *K*_i_ graphs).

**Figure 7:**
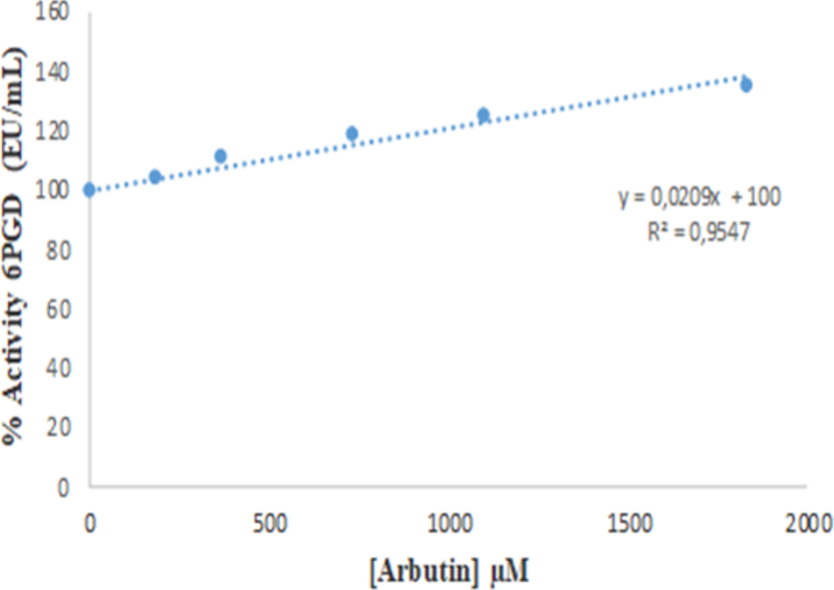
In vitro effect of arbutin on the rat erythrocyte 6PGD enzyme.

In in vitro studies, the 6PGD enzyme, which catalyzes the third step of the oxidative reactions of the pentose phosphate pathway and shows structural similarity with the G6PD enzyme, was used to purify rat erythrocytes. In vitro study results showed that potassium bromate ions inhibited 6PGD enzyme with IC_50_ = 346 μM with the help of % activity-KBrO_3_ inhibitor concentration graph. When the evaluation of the Lineweaver–Burk graph created with three various inhibitor doses was performed, it was found that potassium bromate ions inhibited the 6PGD enzyme with *K*_i_ = 434 μM ± 6.17 noncompetitively (Figure 6). It was determined that arbutin activates the 6PGD enzyme purified from rat erythrocytes by affinity chromatography under in vitro conditions (Figure 7).

## Discussion

Arbutin is chemically composed of 4-hydroxyphenyl-*b*-glucopyranoside. This hydroquinone derivative is mainly biosynthesized by *Ericaceae* and *Saxifragaceae* species. In phytotherapy, arbutin extracts from the leaves of *Arctostaphyllos uva ursi* (*Ericaceae*) have been employed in phytotherapy. Arbutin and hydroquinone prevent melanin production; they have been shown to inhibit the activity of tyrosinase. Due to this, it is employed as a common skin-lightening agent in the treatment of hyperpigmentation.^32^ Hydroquinones, which are arbutin aglycons, also show antibacterial and antioxidant effects.^22^ Potassium bromate, which is used as a food additive, causes DNA damage, especially in the liver and intestines, causing respiratory disorders and asthma. Previous research shows that potassium bromate causes kidney, stomach, thyroid, and intestinal cancer in animals.^1^ For the prevention of potassium bromate toxicity, a definitive treatment method has not been developed until recently.

The pentose phosphate pathway, one of the primary pathways of carbohydrate metabolism, mainly produces NADPH and Ribose 5-phosphate (R5P) molecules. NADPH produced in the pentose phosphate pathway is used in intracellular reductive biosynthesis reactions, reactive oxygen species (ROS) scavenging, and biosynthesis of lipids. R5P is used in the biosynthesis of nucleotides necessary for both cell division and proliferation. The production of nucleotides uses R5P which is necessary for both cell division and proliferation. Inhibition of G6PD and 6PGD enzymes in normal cells weakens the antioxidant defense of cells against damage by oxidized molecules. It disrupts the biosynthesis of lipids, constitutive molecules for cell membranes. Therefore, cell proliferation and apoptosis also cause inhibition.^33^

In this study, the potential ameliorative effect of arbutin, a natural compound found in plants, on potassium bromate-induced enzyme inhibition in erythrocyte tissue was investigated. When the results of the study were analyzed, it was shown that the potassium bromate-treated rats showed a significant inhibition of the G6PD enzyme activity compared to the rats in the control group. While it was shown that the arbutin group’s enzyme activity increased. The enzyme activity in the potassium bromate + arbutin group was determined to be similar to that in the control group. When the change in 6PGD enzyme activity was analyzed, it was determined that the enzyme activity decreased in the potassium bromate group compared to the control group, and this decrease was statistically significant. It was discovered that the arbutin group had levels of enzyme activity that were greater than those of the control group. In the potassium bromate + arbutin group, on the other hand, compared to the potassium bromate group, the 6PGD enzyme activity significantly increased, potassium bromate’s inhibitory impact was lessened, and the 6PGD enzyme activity became closer to that of the control group. The first and third steps of the oxidative processes in the pentose phosphate pathway are catalyzed by the regulating enzymes G6PD and 6PGD.

Glutathione (GSH) is vital for many metabolic reactions such as the protection and regulation of the thiol-redox state of the cell, the protection of the cell against the damage of oxidized molecules, the regulation of apoptotic cell death, the regulation of caspase activity, the activation of transcription factors, Bcl-2 expression, and ceramide production.^34^ Because of these important reactions, GSH is related to cardiovascular diseases, aging, cystic fibrosis, and various immune diseases, especially cancer.^35^ GR and GST enzymes are important enzymes involved in GSH metabolism.^13^ According to the findings of our study, the potassium bromate group’s GR enzyme activity was significantly decreased, whereas the arbutin group’s GR enzyme activity increased in comparison to the control group. In the potassium bromate + arbutin group, although the GR activity was higher than that in the potassium group, the enzyme was found to be inhibited compared to the control group. Therefore, arbutin was found to be insufficient in reducing the level of GR enzyme inhibition. In the potassium bromate group, the GST enzyme activity was close to that of the control group. This result showed that potassium bromate had no inhibitory effect on the GST enzyme activity. In comparison to the control group, the arbutin group was shown to have a greater GST enzyme activity.

Thioredoxin protein (Trx), NADPH, and thioredoxin reductase enzyme together form the thioredoxin system. The thioredoxin system plays a critical role in many biochemical mechanisms that are vital for the cell. Trx1 degrades ribonucleotide reductase (RNR). Through this reaction, it provides the formation of deoxyribose sugars, which are necessary for DNA synthesis from ribose sugars. The Trx system is also effective in binding many transcription factors such as p53, AP-1, and the glucocorticoid receptor to DNA. Trx protects the cell against oxidative stress by providing electrons to peroxidases and removing hydrogen peroxide and peroxynitrite.^21,36^ Several diseases, including cancer, diabetes, cardiovascular and neurological disorders, and rheumatoid arthritis have been linked to the Trx system due to these important reactions in the cell.^37,38^ The findings of this investigation demonstrated that potassium bromate ions inhibited TrxR activity in vivo, increased the enzyme activity of arbutin, and decreased the inhibition effect caused by potassium bromate ions.

The 6PGD enzyme was purified from rat erythrocyte tissues in the in vitro phase of the investigation, using hemolysate preparation and 2′,5′ ADP Sepharose-4B affinity chromatography. Then, the effect of different concentrations of potassium bromate ions on the pure enzyme activity was investigated spectrophotometrically. In order to calculate the inhibitory concentration (IC_50_), which is the level at which the 6PGD enzyme activity is 50% inhibited, % activity-inhibitor concentration graph was drawn. With the help of the prepared % activity-KBrO_3_ concentration chart, potassium bromate ions were determined to inhibit the enzyme with IC_50_ = 346 μM. In the last stage of the study, it was discovered that potassium bromate ions inhibited the 6PGD enzyme noncompetitively with *K*_i_ = 434.4 μM ± 6.1 by the Lineweaver–Burk graph. These results showed that the in vivo and in vitro results of our study were consistent with each other.

Previous research focused on the effects of arbutin and potassium bromate ions on enzymes. In a study conducted by Oliver et al., the effect of arbutin on phospholipase A enzymes was investigated. In this study, liposomes of different compositions were lyophilized in the presence and absence of phospholipase enzymes. Study results showed that when liposomes were hydrated at 76%, arbutin inhibited PLA2 but did not affect phospholipase B or C.^39^ In a study conducted by Nadi et al., the radioprotective effect of arbutin in mice exposed to megavoltage therapeutic X-ray was investigated using serum alkaline phosphatase (ALP), alanine aminotransferase (ALT), and aspartate aminotransferase (AST) activity measurements. Study results showed increased ALT, ALP, and AST activity levels on days 1 and 7 after irradiation. It also showed that arbutin, whole body X irradiation (2 or 4 Gy) caused a significant decrease in ALT and ALS levels compared to other groups over time intervals and could regulate the ALP level. Based on this result, it was reported that arbutin is a powerful radioprotector that reduces the radiation effect on all body tissues.^40^ Ahmad et al. investigated the potential role of taurine in protecting against KBrO_3_-induced kidney damage in rats. The results of the research showed that potassium bromate ions reduced enzyme activities (35–65%) such as leucine aminopeptidase, alkaline phosphatase, γ-glutamyl transferase in the kidney cortex and medulla tissue.^41^

## Conclusions

In conclusion, this study investigated the inhibition effect of potassium bromate, a food additive, on the pentose phosphate pathway’s enzymes as well as the antioxidant systems of glutathione and thioredoxin which are vital for metabolism. It also investigated the potential of arbutin to reduce this effect. The study’s findings demonstrated that potassium bromate ions inhibited enzyme activities both in vivo and in vitro, whereas arbutin regulated enzyme activities by lowering this inhibitory impact. These results will be enlightening the understanding of the pathophysiology of metabolic disorders caused by potassium bromate in the future.

## Data Availability

All data generated or analyzed during this study are included in this published article.

## Notes

The study was started after approval of the experimental procedures by the Bingöl University Animal Experiments Local Ethics Committee (BUHADYEK: 12.07.2023-E.113967).
